# Identification of Tumor Microenvironment and DNA Methylation-Related Prognostic Signature for Predicting Clinical Outcomes and Therapeutic Responses in Cervical Cancer

**DOI:** 10.3389/fmolb.2022.872932

**Published:** 2022-04-19

**Authors:** Bangquan Liu, Jiabao Zhai, Wanyu Wang, Tianyu Liu, Chang Liu, Xiaojie Zhu, Qi Wang, Wenjing Tian, Fubin Zhang

**Affiliations:** ^1^ Department of Epidemiology, College of Public Health, Harbin Medical University, Harbin, China; ^2^ Department of Gynecological Oncology, Harbin Medical University Cancer Hospital, Harbin, China

**Keywords:** tumor microenvironment, DNA methylation, prognostic model, drug response, immunotherapy response, cervical cancer

## Abstract

**Background:** Tumor microenvironment (TME) has been reported to have a strong association with tumor progression and therapeutic outcome, and epigenetic modifications such as DNA methylation can affect TMB and play an indispensable role in tumorigenesis. However, the potential mechanisms of TME and DNA methylation remain unclear in cervical cancer (CC).

**Methods:** The immune and stromal scores of TME were generated by the ESTIMATE algorithm for CC patients in The Cancer Genome Atlas (TCGA) database. The TME and DNA methylation-related genes were identified by the integrative analysis of DNA promoter methylation and gene expression. The least absolute shrinkage and selection operator (LASSO) Cox regression was performed 1,000 times to further identify a nine-gene TME and DNA methylation-related prognostic signature. The signature was further validated in Gene Expression Omnibus (GEO) dataset. Then, the identified signature was integrated with the Federation International of Gynecology and Obstetrics (FIGO) stage to establish a composite prognostic nomogram.

**Results:** CC patients with high immunity levels have better survival than those with low immunity levels. Both in the training and validation datasets, the risk score of the signature was an independent prognosis factor. The composite nomogram showed higher accuracy of prognosis and greater net benefits than the FIGO stage and the signature. The high-risk group had a significantly higher fraction of genome altered than the low-risk group. Eleven genes were significantly different in mutation frequencies between the high- and low-risk groups. Interestingly, patients with mutant *TTN* had better overall survival (OS) than those with wild type. Patients in the low-risk group had significantly higher tumor mutational burden (TMB) than those in the high-risk group. Taken together, the results of TMB, immunophenoscore (IPS), and tumor immune dysfunction and exclusion (TIDE) score suggested that patients in the low-risk group may have greater immunotherapy benefits. Finally, four drugs (panobinostat, lenvatinib, everolimus, and temsirolimus) were found to have potential therapeutic implications for patients with a high-risk score.

**Conclusions:** Our findings highlight that the TME and DNA methylation-related prognostic signature can accurately predict the prognosis of CC and may be important for stratified management of patients and precision targeted therapy.

## Introduction

Cervical cancer (CC) is the fourth leading cause of cancer-related death in women, with more than 300,000 deaths worldwide each year (Cohen et al., 2019), of which adenocarcinoma, squamous cell carcinoma, and adenosquamous carcinoma are common pathological types (Small et al., 2017). The incidence of CC is gradually declining due to the identification of HPV as a causative factor and the introduction of specific vaccines into clinical practice (Wakeham and Kavanagh, 2014; Herrero et al., 2015; Ogilvie et al., 2018). Although goals have been achieved in preventing CC, when patients are diagnosed at an advanced stage, the prognosis is extremely poor, with 5-year overall survival (OS) less than 40% (Lin et al., 2010). Currently, immunotherapy is one of the best treatment strategies for patients with advanced CC (Wendel Naumann and Leath, 2020). However, tumor heterogeneity makes it difficult to accurately assess the prognosis of each patient after immunotherapy, which is also a shortcoming of the Federation International of Gynecology and Obstetrics (FIGO) stage system (Wright et al., 2019). Therefore, accurate molecular predictors are needed to improve the prediction of CC prognosis and guide the individual evaluation of immunotherapy, especially those at high risk of recurrence or death.

Tumor microenvironment (TME) is defined as the environment surrounding the tumor, including various immune cells, stromal cells, extracellular matrix molecules, and cytokines, among which immune cells and stromal cells are closely related to tumor progression and treatment outcome (Hanahan and Coussens, 2012; Binnewies et al., 2018), and the genetic and epigenetic modifications acquired by the TME also play important roles in tumorigenesis and lead to uncontrolled growth of tumor cells (Sharma et al., 2010). Among all epigenetic modifications, DNA methylation is a stable change in gene structure and is one of the most studied mechanisms involved in regulating gene expression (Bird, 2007). DNA hypermethylation in the promoter region of genes encoding inhibitory immune checkpoints, tumor suppressors, and suppressive cytokines can lead to impaired activation of anti-tumor immunity, immune escape, drug resistance, tumor growth, and TME dyshomeostasis and significantly promote the development and progression of cancer (Easwaran et al., 2014; Ali et al., 2017).

In this study, we calculated immune and stromal scores based on the ESTIMATE algorithm to estimate the TME status of each CC patient and found that the immune scores were associated with patients’ prognoses. We correlated epigenetic characteristics and TME status by analyzing the multi-omics data (RNA sequencing and DNA methylation array) across different immune groups and identified the TME and DNA methylation-related prognostic signature. We then used microarray data from the Gene Expression Omnibus (GEO) database for validation. Both the developed signature and the nomogram based on the signature and FIGO stage showed high potential for individual risk stratification and prognosis prediction. Furthermore, we sought to understand the relationship between the signature and tumor mutation status, genetic variants, and pathway activation. Finally, we not only identified four agents for these high-risk score patients but also assessed the role of this signature in identifying immune responders to immunotherapy. The results gathered from this study may be valuable in predicting patients’ prognosis and facilitating the individualization of immune treatment strategies for CC.

## Materials and Methods

### Data Acquisition and Processing

The Cancer Genome Atlas (TCGA) RNA-seq data, Illumina 450k DNA methylation data, somatic mutation data, copy number variation data, and clinical datasets of 306 CC patients were downloaded from Genomic Data Commons Data Portal (https://portal.gdc.cancer.gov/). FPKM values were transformed into transcripts per kilobase million (TPM) values. Quantile normalized microarray gene expression data and clinical annotations of GSE44001 were obtained from the GEO database (https://www.ncbi.nlm.nih.gov/geo/). All samples with a survival time of 0 or duplicates were deleted, and TCGA 291 samples and GEO 283 samples were used for further analysis. Expression profile data of human cancer cell lines (CCLs) were obtained from the Broad Institute Cancer Cell Line Encyclopedia (CCLE) project (https://portals.broadinstitute.org/ccle/) (Ghandi et al., 2019). The sensitivity data were obtained from the Cancer Therapeutics Response Portal (CTRP v.2.0, released October 2015, https://portals.broadinstitute.org/ctrp) and PRISM Repurposing dataset (19Q4, released December 2019, https://depmap.org/portal/prism/), respectively. In the two datasets, drug sensitivity is measured using the area under the curve (AUC) value and a lower AUC value indicates increased treatment sensitivity. The compounds with more than 20% of missing data were removed, and K-nearest neighbor (k-NN) imputation was used to impute the missing AUC values (Yang et al., 2021).

### Differential Expression Genes (DEGs) and Differential Methylation Genes (DMGs) Analysis

Limma analysis (Ritchie et al., 2015) was carried out to identify DEGs between low- and high-immune score groups. The genes meeting the |log_2_FC| > 1.0 and adjusted *p*-value < 0.05 were considered as DEGs. DNA methylation level for each gene was estimated by calculating the average beta value of probes in promoter regions including TSS200, 1stExon, TSS1500, and 5′UTR (Jiao et al., 2014). An unpaired *t*-test was performed to identify DMGs between low- and high-immune score groups. The *p-*value was adjusted by the Benjamini Hochberg method. DMGs were defined by |log_2_FC| > 0.1 and the false discovery rate corrected *p*-value < 0.05.

### Correlation Analysis Between DNA Promoter Methylation and Genes

The Pearson correlation (r) was calculated between the mean β values of the DNA promoter region and the normalized expression values of the corresponding genes to examine the effect of DNA methylation in the promoter region on gene expression levels. Cut-off for significant correlations was set at |r| > 0.3 and *p-*value < 0.05 (Piao et al., 2012).

### Identification of the Prognostic Genes and Calculation of the Risk Score

Robust prognostic genes in TCGA CC samples were identified using multi-step processes. First, univariate Cox regression analysis was performed to screen prognosis-related genes, and genes with *p*-value less than 0.05 were selected for further analysis. Next, we used the least absolute shrinkage and selection operator (LASSO) Cox regression analysis to assess the correlation between the gene expression and prognosis. This procedure was repeated 1,000 times, and the genes with 100 repetitions were kept for the next step analysis. Further, the concordance index (C-index) was calculated of each possible threshold from one to the number of genes, and the one (k) genes were selected that could reach the largest C-index in the TCGA cohort as the appropriate threshold of the signature. Then, the selected genes were used to perform multivariate Cox regression.

The risk score was calculated by the formula risk score = Σ βi*Expi, where βi is the coefficient of each gene in the multivariate Cox model and Expi represents the normalized expression value of each gene transformed by log_2_ and z-score. Patients were divided into high- and low-risk groups using the median risk score as the cut-off.

### Construction of Nomogram

Based on the multivariate analysis results, we integrated the FIGO stage and risk signature to construct a composite prognostic model using the Cox proportional hazard regression in the TCGA cohort. Then, the R package “rms” was utilized to generate the nomogram. The consistency between the predicted and actual survival outcomes was assessed using the calibration curves. Moreover, time-dependent C-index and the decision curve analysis (DCA) were performed to compare the predictive accuracy of the nomogram, prognostic signature risk model, and FIGO stage.

### Enrichment Analysis and Tumor Immune Signature Analysis

Differentially expressed genes in CC patients between different risk score groups were analyzed by limma. The log_2_FC value of each gene was used as an input to carry out gene set enrichment analysis (GSEA) (Subramanian et al., 2005). The adjusted *p* < 0.05 was considered significantly enriched. Meanwhile, gene set variation analysis (GSVA) was performed to find significantly associated pathways, and adjusted *p* < 0.01 was considered statistically significant. The gene set “h.all.v7.2.symbols.gmt” was selected as the reference gene set.

Signature-related gene modules in the TCGA expression file were identified by weighted gene co-expression network analysis (WGCNA) (Langfelder and Horvath, 2008). The basic set parameters of the program included setting the scale-free topological fit index (R^2^) > 0.85, the minimum cluster size to 30, and the merge threshold function to 0.3. Gene modules with biweight midcorrelation coefficient (r) ≥ 0.5 and *p*-value < 0.05 were defined as signature-related gene modules.

Immune signatures were evaluated from the gene expression levels of immune checkpoints and human leukocyte antigen (HLA) genes (De Simone et al., 2016; Johnston et al., 2019) and the levels of immune cells infiltrating. The infiltrating immune cells levels were calculated by CIBERSORT (Newman et al., 2015), TIMER (Li et al., 2016), and MCP-counter (Becht et al., 2016) algorithms.

### Somatic Variants Analysis and Copy Number Variation Analysis

Logistic regression analysis was performed to adjust for the influence of other clinical pathological features to identify differential mutation patterns, and genes with *p* < 0.05 were defined as significantly mutant genes. Genes with more than five mutations in at least one group were analyzed. The R package “maftools” (Mayakonda et al., 2018) was used to create the visualization of the mutations.

Genomic identification of significant targets in cancer (GISTIC) analysis was used to analyze the copy number variation data and identify the significant amplification and deletion regions and all gene’s discrete copy number status between different risk groups, which was performed by the GISTIC 2.0 pipeline (GenePattern, https://genepattern.broadinstitute.org/).

### Drug Response Prediction

The CTRP and PRISM datasets were utilized to construct predictive models of drug response. Before subsequent analysis, more than 20% of the compounds containing NAs in the samples were excluded. ISOpure algorithm was utilized to reduce the impact of non-tumor components on analysis results (Anghel et al., 2015). A built-in ridge regression model of the “pRRophetic” package was used to estimate the AUC value of each compound in each patient by inputting TCGA purified expression profile and drug sensitivity data.

### Immunotherapeutic Response Prediction

The Tumor Immune Dysfunction and Exclusion (TIDE) algorithm (Fu et al., 2020) and immunophenoscore (IPS) (Charoentong et al., 2017) were leveraged to predict the clinical response to immunotherapy of different risk groups based on the gene expression profile of TCGA CC samples. Patients with higher IPS and lower TIDE scores responded better to immunotherapy.

### Statistical Analysis

All statistical tests were performed in R statistical software (v3.6.3). Unless otherwise noted, a comparison of a continuous variable in two or more than two groups was performed using Wilcoxon rank-sum test or Kruskal–Wallis test. The correlation between two continuous variables was measured by either Pearson’s (r) correlation coefficient or Spearman’s rank-order correlation. Immune and stromal scores were estimated to the TCGA cohort using the ESTIMATE algorithm (Yoshihara et al., 2013). Kaplan–Meier (KM) survival analysis was used to assess prognosis between different groups by the log-rank test in the “survival” R package. The time-dependent AUC was performed using the “timeROC” R package. The time-dependent C-index was performed using the “pec” R package. The *p*-value is two-sided, and *p* < 0.05 was considered statistically significant.

## Results

### Overview of Workflow

The whole workflow of this study was delineated in Supplementary Figure S1, including the identification of hypermethylated-downregulated genes; development and validation of the prognostic signature; the construction of the nomogram; and the analyses of signature-associated immune signature, function enrichment, and genetic features.

### Analysis of the Correlation of Immune and Stromal Scores With Clinical Features

The detailed clinical information of patients in the TCGA cohort is shown in Supplementary Table S1. Immune scores ranged from −1,203.5 to 3,430.4, and stromal scores of these patients ranged from −2,433.1 to 812.7. The median cut-off values of immune scores and stromal scores were applied to stratify CC patients into high- and low-immune groups and high- and low-stromal groups. KM analysis result showed that the OS of patients in the high-immune group was better than that of the low-immune group, but there was no difference in OS between the high- and low-stromal groups (Supplementary Figures S2A,B). We also found a strong negative correlation between immune score and tumor purity (Supplementary Figure S2C). However, immune scores were not associated with the FIGO stage (Supplementary Figure S2D) and the tumor node metastasis (TNM) stage (Supplementary Figures S2E–G).

### Identification of DEGs and DMGs Between High- and Low-Immune Groups

**FIGURE 1:**
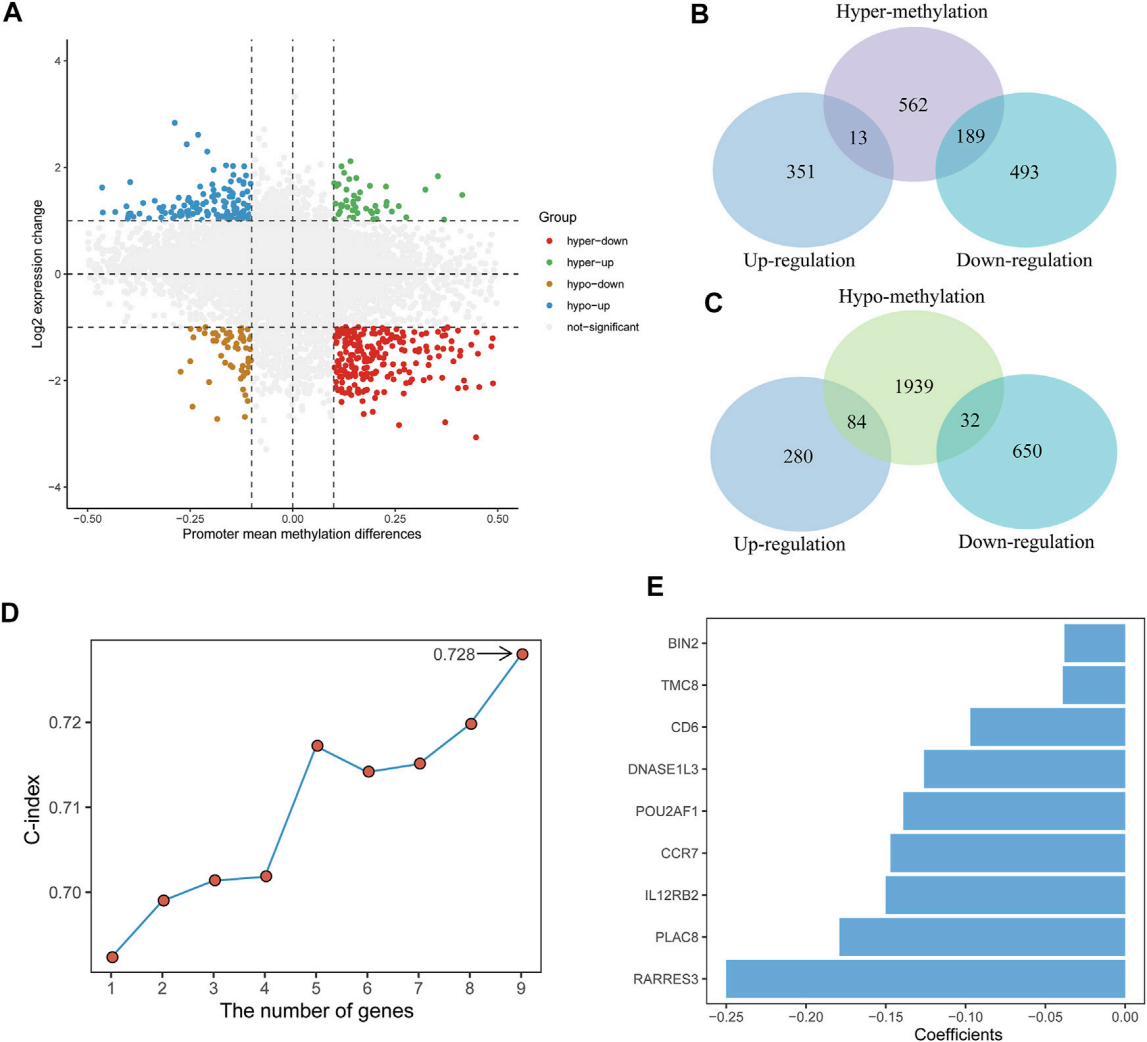
Identification of the TME and DNA methylation-related prognostic signature. **(A)** Scatter plot of promoter mean methylation difference and gene expression levels change. hyper-up, hypermethylated-upregulated; hyper-down, hypermethylated-downregulated; hypo-up, hypomethylated-upregulated; hypo-down, hypomethylated-downregulated. **(B,C)** Venn diagrams showing the intersection between DEGs and hypermethylated genes (top) and between DEGs and hypomethylated genes (bottom). **(D)** The C-index of different genes combinations in the signature. **(E)** The nine genes included in the signature. Corresponding coefficients are depicted by horizontal bars.

We mapped the average β value of the DNA promoter region to genes, and 14,932 genes were obtained (Figure 1A). Differential methylation and expression analyses were carried out between high- and low-immune groups. A total of 2,819 DMGs were detected, with 764 hypermethylated genes and 2,055 hypomethylated genes. A total of 1,046 DEGs were detected, with 364 upregulated genes and 682 downregulated genes.

The integrative analysis of gene expression and DNA promoter methylation in CC patients was performed by identifying the intersection between the DEGs and DMGs. Of the 764 hypermethylated genes, 13 genes were upregulated and 189 genes were downregulated (Figure 1B). Among the 2,055 hypomethylated genes, 84 genes were upregulated and 32 genes were downregulated (Figure 1C). Then, we focused on the hypermethylated-downregulated genes and used the Pearson correlation analysis to examine the impact of DNA promoter methylation on gene expression. Among the 189 hypermethylated-downregulated genes, 111 genes revealed significantly negative correlations (Supplementary Table S2), and mRNA expression of these genes is shown in Supplementary Figure S3.

### Identifying Prognostic Genes and Development of the Risk Score

A total of 291 TCGA CC patients with available clinical information were used to recognize the prognostic signature. We first used univariate Cox proportional hazards regression analysis and identified 55 genes correlated with OS (*p* < 0.01) (Supplementary Table S3). After a 1,000-time LASSO Cox regression analysis, we identified nine genes (*CCR7*, *CD6*, *POU2AF1*, *TMC8*, *PLAC8*, *RARRES3*, *BIN2*, *DNASE1L3*, and *IL12RB2*) that were stably associated with prognosis over 100-time iterations (Supplementary Table S4).

For all possible thresholds from 1 to 9, a nine-gene set with the largest C-index (0.728) was considered prognosis-associated genes (Figure 1D, Supplementary Table S5). All nine genes showed a high negative correlation between DNA promoter mean methylation and gene expression (Supplementary Figure S4). Furthermore, we estimated the risk score based on the linear combination of the nine-gene expression levels weighted by their multivariate Cox regression coefficients (Figure 1E): risk score = (−0.147) ✕ *CCR7* + (−0.097) ✕ *CD6* + (−0.139) ✕ *POU2AF1* + (−0.039) ✕ *TMC8* + (−0.179) ✕ *PLAC8* + (−0.250) ✕ *RARRES3* + (−0.038) ✕ *BIN2* + (−0.126) ✕ *DNASE1L3* + (−0.150) ✕ *IL12RB2*. Then, according to the median risk score, CC patients were divided into low-risk (*n* = 145) and high-risk groups (*n* = 146).

### The Prognostic Value of Risk Score

**FIGURE 2:**
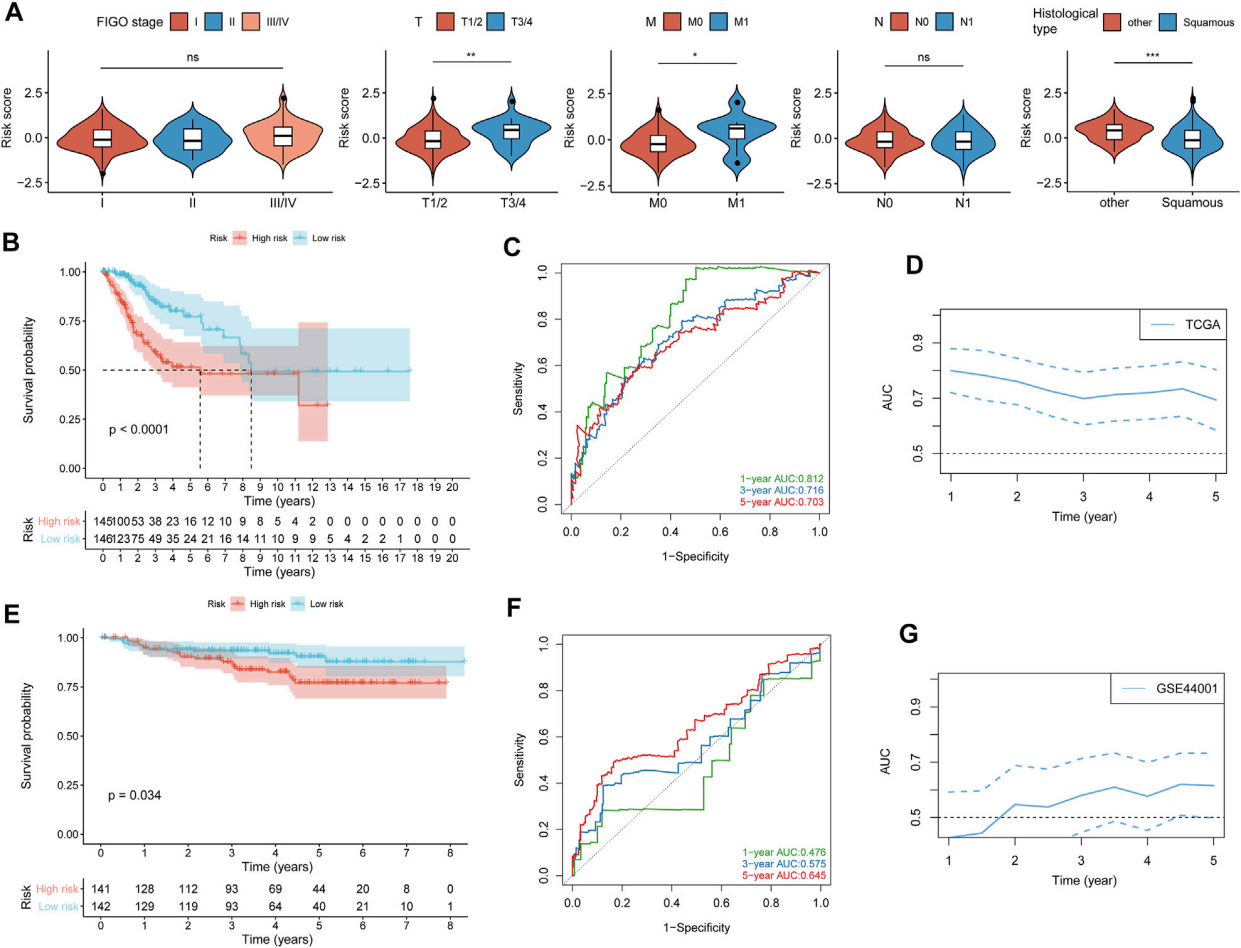
Validation of the prognostic value of the risk score. **(A)** Difference analysis of the distribution of risk scores in different FIGO stages, TNM stages, and histological types. **(B)** Kaplan–Meier curves for differential detection of patients in the TCGA cohort by the log-rank test. **(C)** ROC curves of risk scores used to predict 1-year, 3-year, and 5-year survival in the TCGA cohort. **(D)** Time-dependent ROC curves of the risk score in the TCGA cohorts. **(E)** Kaplan–Meier curves for differential detection of patients in the GSE44001 cohort by the log-rank test. **(F)** ROC curves of risk scores used to predict 1-year, 3-year, and 5-year survival in the GSE44001 cohort. **(G)** Time-dependent ROC curves of the risk score in the GSE44001 cohorts. *p < 0.05; **p < 0.01; ***p < 0.001; ns, not significant.

A heatmap of expression levels of the nine identified genes and the scatterplot of OS with a corresponding risk score are illustrated in Supplementary Figure S5A. We explored the distribution of the risk score with histological type, TNM stage, and FIGO stage. Patients with a higher M stage and T stage had a higher risk score, and patients in the squamous subtype had a significantly lower risk score than those in other subtypes (Figure 2A). We next found that patients with low-risk scores were significantly associated with better OS compared with patients with high-risk scores (Figure 2B). Moreover, the accuracy of the risk score in OS prediction was evaluated using the AUC, as shown in Figures 2C,D. The AUCs of the risk score model at 1, 3, and 5 years were 0.812, 0.716, and 0.703, respectively.

To confirm that the risk score had a stable prognostic value across different datasets, we corroborated this association in an external validation GEO (GSE44001) dataset. A heatmap of the signature consisting of nine genes and the scatterplot of disease-free survival (DFS) time with corresponding risk score in GEO (GSE44001) are shown in Supplementary Figure S5B. Consistent with the above TCGA results, patients with high-risk scores in GSE44001 had a significantly poorer DFS than those with low-risk scores (Figure 2E), and AUCs at 1, 3, and 5 years were 0.476, 0.575, and 0.645, respectively (Figures 2F,G). The result showed that the risk score did not show high accuracy in predicting the prognosis of CC patients in the validation dataset (GSE44001), which may be caused by the CC patients in the early stage (stages I-II).

**FIGURE 3:**
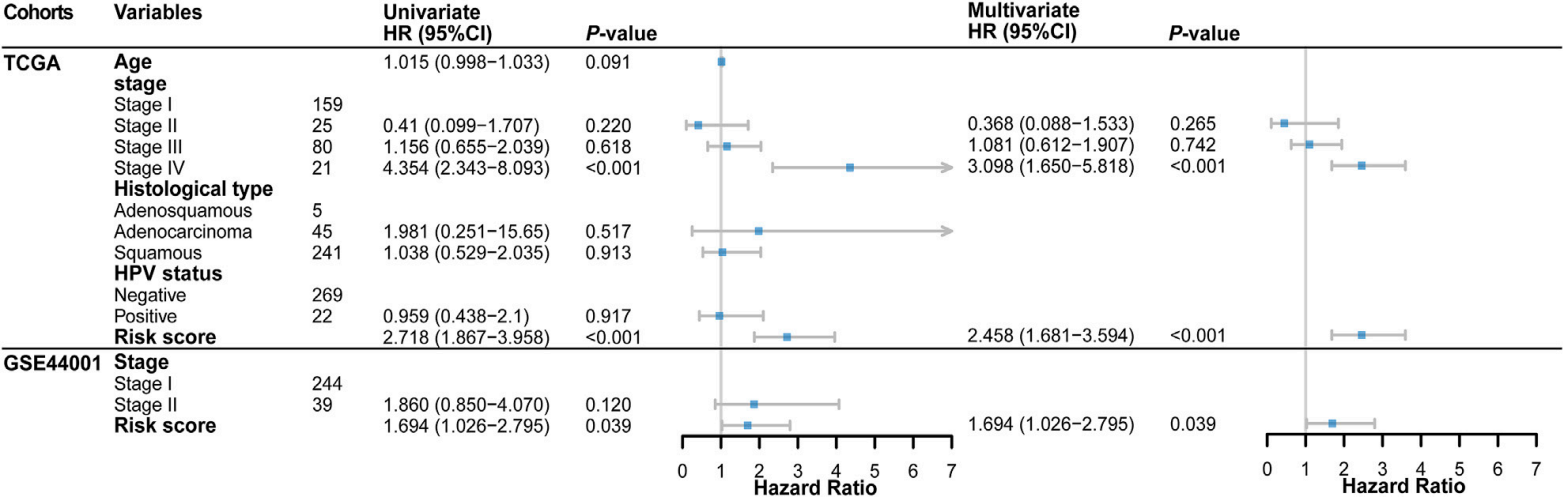
Forest plot of the univariate and multivariate Cox regression analysis in TCGA and GSE44001 cohorts.

The results of univariate and multivariate Cox regression analysis further showed that risk score could be an independent predictor of survival outcome in CC patients after being adjusted for the clinicopathological features (Figure 3), suggesting that the TME and DNA methylation-related genes might be involved in CC occurrence and development and could serve as potential therapeutic targets. Meanwhile, we also found that the tumor FIGO stage could be used as an independent predictor.

### Construction of Nomogram

**FIGURE 4:**
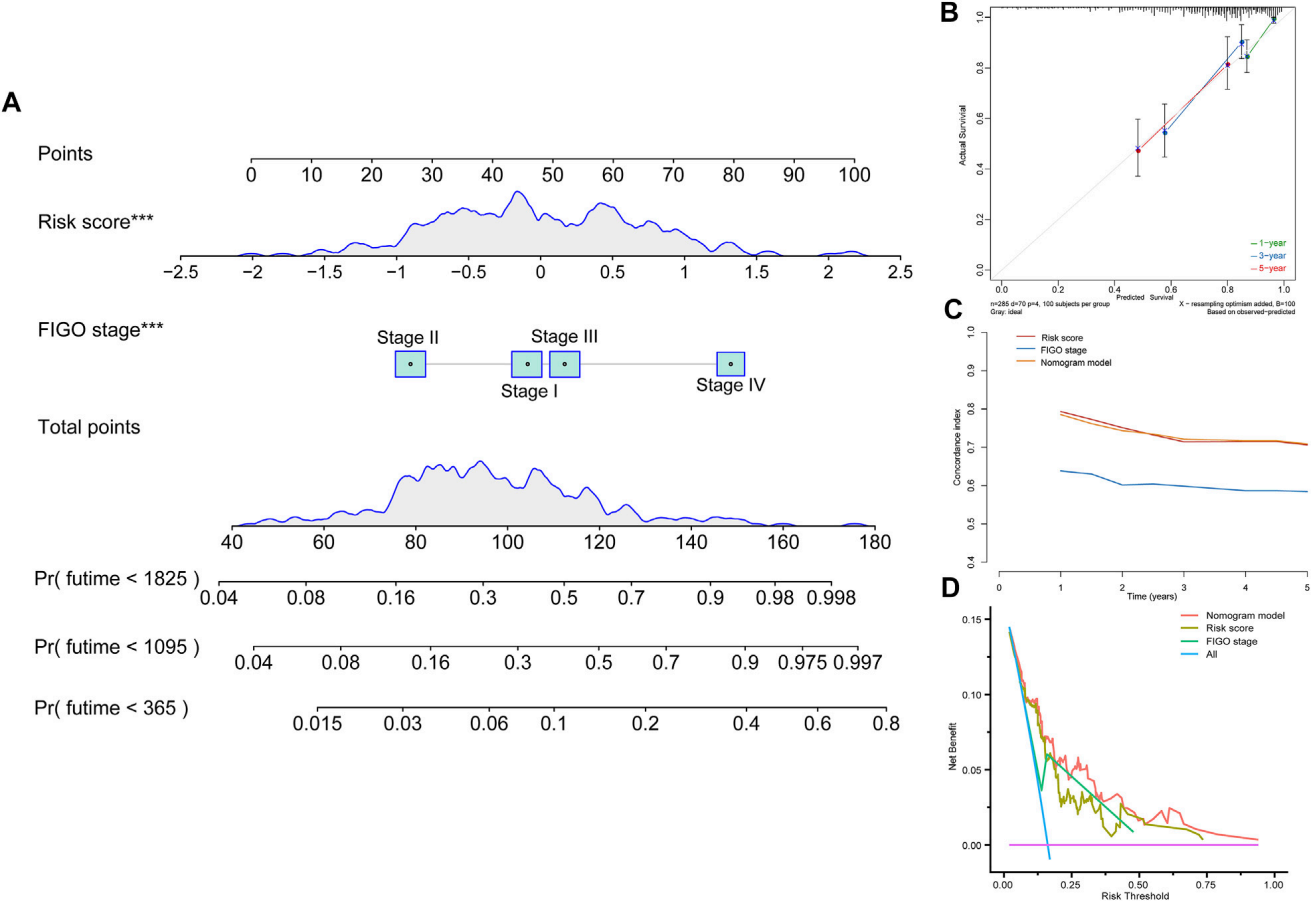
Construction of a nomogram model. **(A)** Nomogram constructed in conjunction with the risk score and FIGO stage for the TCGA cohort. **(B)** Calibration plot of the nomogram. **(C)** C-index curves of the FIGO stage, risk score, and nomogram. **(D)** Decision curve analysis for evaluating the net benefits of FIGO stage, risk score, and nomogram.

To find a more effective method to strongly predict the prognosis of CC patients, we combined tumor FIGO stage and risk score to establish a complete evaluation signature. A nomogram was created to predict the 1-, 3-, and 5-year prognostic survival probabilities of patients with CC (Figure 4A). The calibration curve was used to assess the consistency between the actual survival status and the predicted outcomes of CC patients (Figure 4B). The result revealed that based on the FIGO stage and risk score, the nomogram could effectively predict the prognosis. Then, we calculated the C-index to confirm this (Figure 4C). These results suggested that the ability of the nomogram to predict the prognosis of CC patients is more reliable than a single independent factor. Moreover, the DCA diagram showed that the net benefits of the nomogram were significantly higher than the risk score and FIGO stage, indicating the good clinical applicability of the nomogram (Figure 4D).

### Risk Score Was Associated With Immune Signature

**FIGURE 5:**
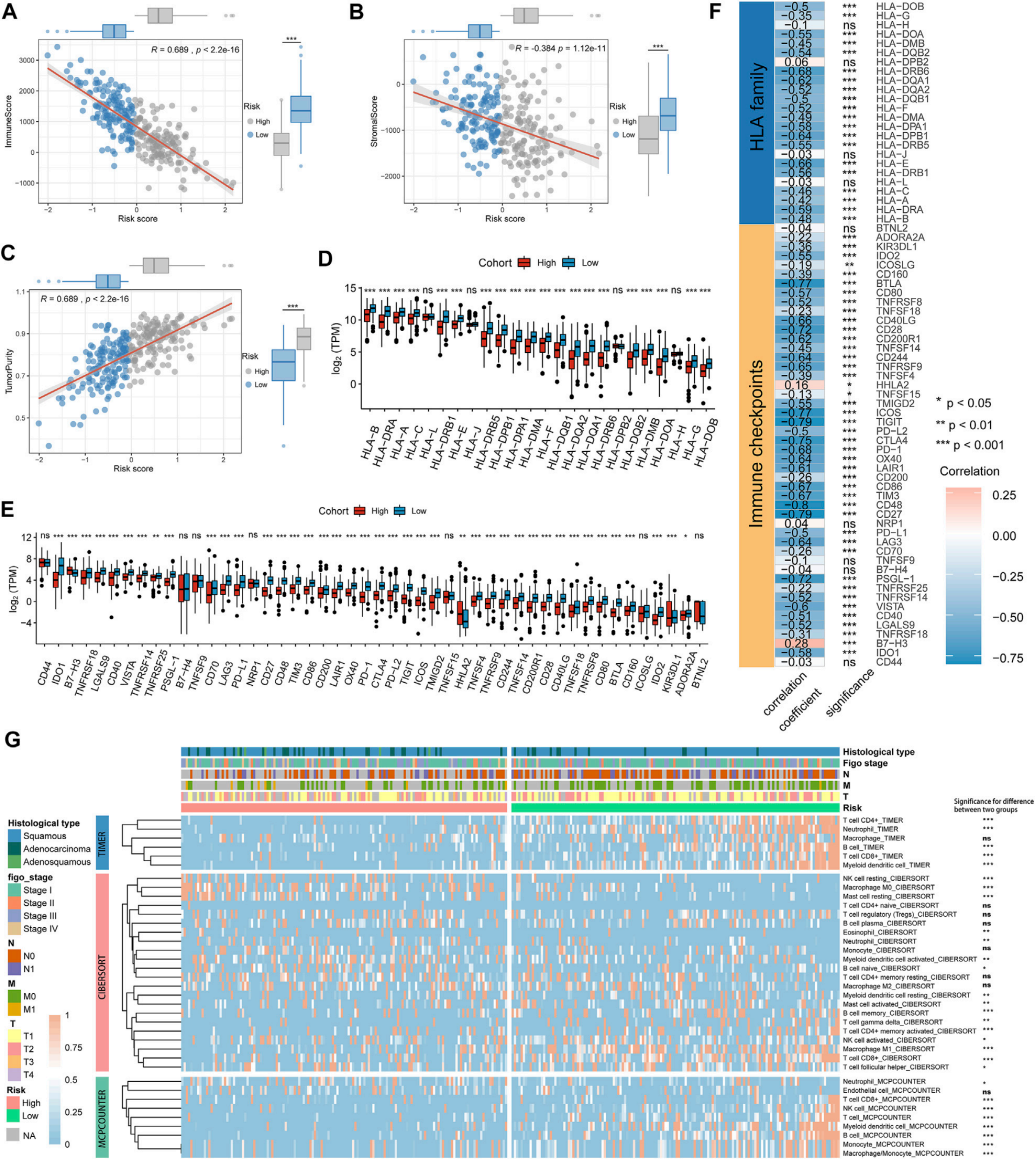
The immune signature between the high- and low-risk groups in the TCGA cohort. **(A–C)** Association between immune score, stromal score, tumor purity, and risk score and their distribution in different risk groups. **(D,E)** Differential analysis of gene expression levels of HLA family genes and immune checkpoints in different risk groups. **(F)** Correlation analysis for the risk score and the gene expression levels of HLA family genes and immune checkpoints. **(G)** The heatmap showing the immune and stromal cell infiltration levels and differences in distribution between different risk groups. *p < 0.05; **p < 0.01; ***p < 0.001; ns, not significant.

To elucidate the interrelation of the risk score and immune signature, we examined the correlation between the risk score and immune and stromal scores, HLA family genes, immune checkpoints, and infiltrating immune cells. The results showed that immune and stromal scores were significantly positively correlated with risk scores. Patients in the low-risk score group had higher immune and stromal scores than those in the high-risk score group, and patients in the low-risk score group had lower tumor purity (Figures 5A−C). We next found that the gene expression levels of 20 HLA family genes and 41 immune checkpoints were significantly different between the high- and low-risk groups (Figures 5D,E, Supplementary Table S6), and the risk score was significantly negatively correlated with the expression levels of 20 HLA genes and 43 immune checkpoints, such as *HLA-DOA*, *HLA-DPB1*, *IDO2*, *BTLA*, and *CD27* (Figure 5F, Supplementary Table S7). TIMER, CIBERSORT, and MCP-counter were performed to estimate the distribution of infiltrating immune cells between the low- and high-risk score groups. Most immune cells and stromal cells were infiltrated more frequently in the low-risk score group. However, antigen presenting cells such as macrophage M0 and T cell regulatory (Tregs) increased in the high-risk score group (Figure 5G, Supplementary Table S8). These results indicate that the suppression of stromal and immune components in the tumor microenvironment likely contributes to the worse prognosis in high-risk patients.

### Function Analysis of Genes Related to the Risk Score

**FIGURE 6:**
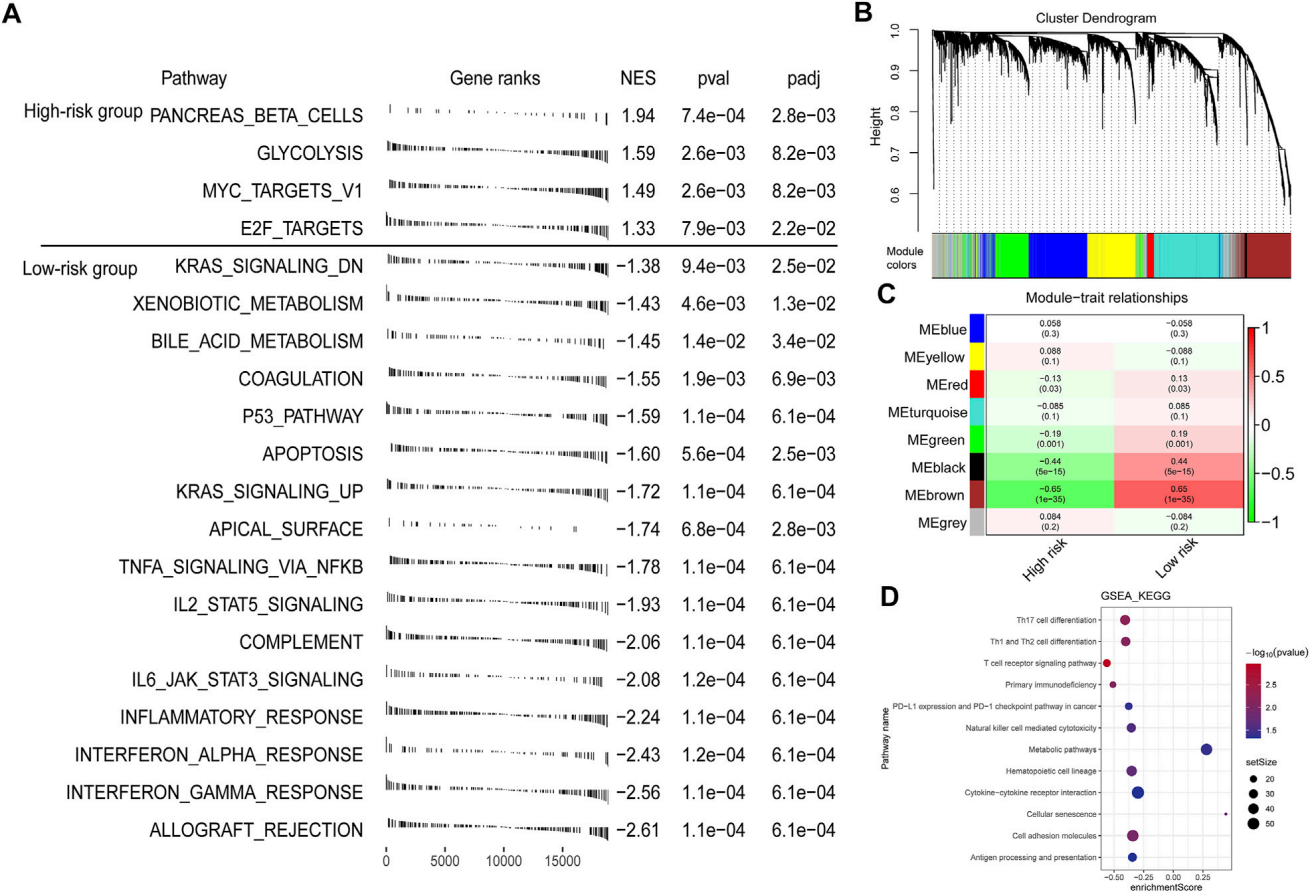
Function analysis of genes correlated with the risk score. **(A)** GSEA enrichment plots showing enriched gene sets against to hallmark dataset in high- and low-risk groups. NES, normalized enrichment score. **(B)** A dendrogram of the top 5,000 genes with the most variation clustered based on the topological overlap together. **(C)** The heatmap showing the association between gene modules and the signature risk score. **(D)** GSEA annotated by KEGG gene sets for the brown module genes.

To explore the underlying mechanisms that lead to different outcomes between the high- and low-risk score groups, we carried out GSEA using annotations of hallmark gene sets. Significantly enriched pathways with adjusted *p*-value < 0.05 are shown in Figure 6A. Genes involved in glycolysis, Myc targets v1, and E2F targets signaling pathway were enriched in the high-risk score group, while genes related to apoptosis, KRAS signaling up, inflammatory response, and p53 signaling pathway were enriched in the low-risk score group.

Furthermore, we performed WGCNA to get the signature-related modules. Based on the median absolute deviation (MAD), the top 5,000 genes with the most variation were selected and the gene expression file of these genes was inputted into the WGCNA. When the lowest soft threshold power was four, the scale-free R2 reached 0.85 (Supplementary Figure S6). We constructed a cluster dendrogram with the adjacency matrix; eight-color modules (blue, yellow, red, turquoise, green, black, brown, and grey) were identified (Figure 6B). Next, we analyzed the module-trait relationships and found that the brown module was highly significantly correlated with the signature risk score (|r| > 0.5) (Figure 6C). We then performed GSEA using the annotations of the KEGG gene set to explore the biological functions of genes in different modules. For brown module genes, the top enriched terms were Th1 and Th2 cell differentiation, T cell receptor signaling pathway, primary immunodeficiency, and PD-L1 and PD-1 checkpoint pathway in cancer, indicating that genes in the brown module are involved in regulating immune system function (Figure 6D).

### Differences in Genetic Variation and Pathway Activation Between High- and Low-Risk Groups

**FIGURE 7:**
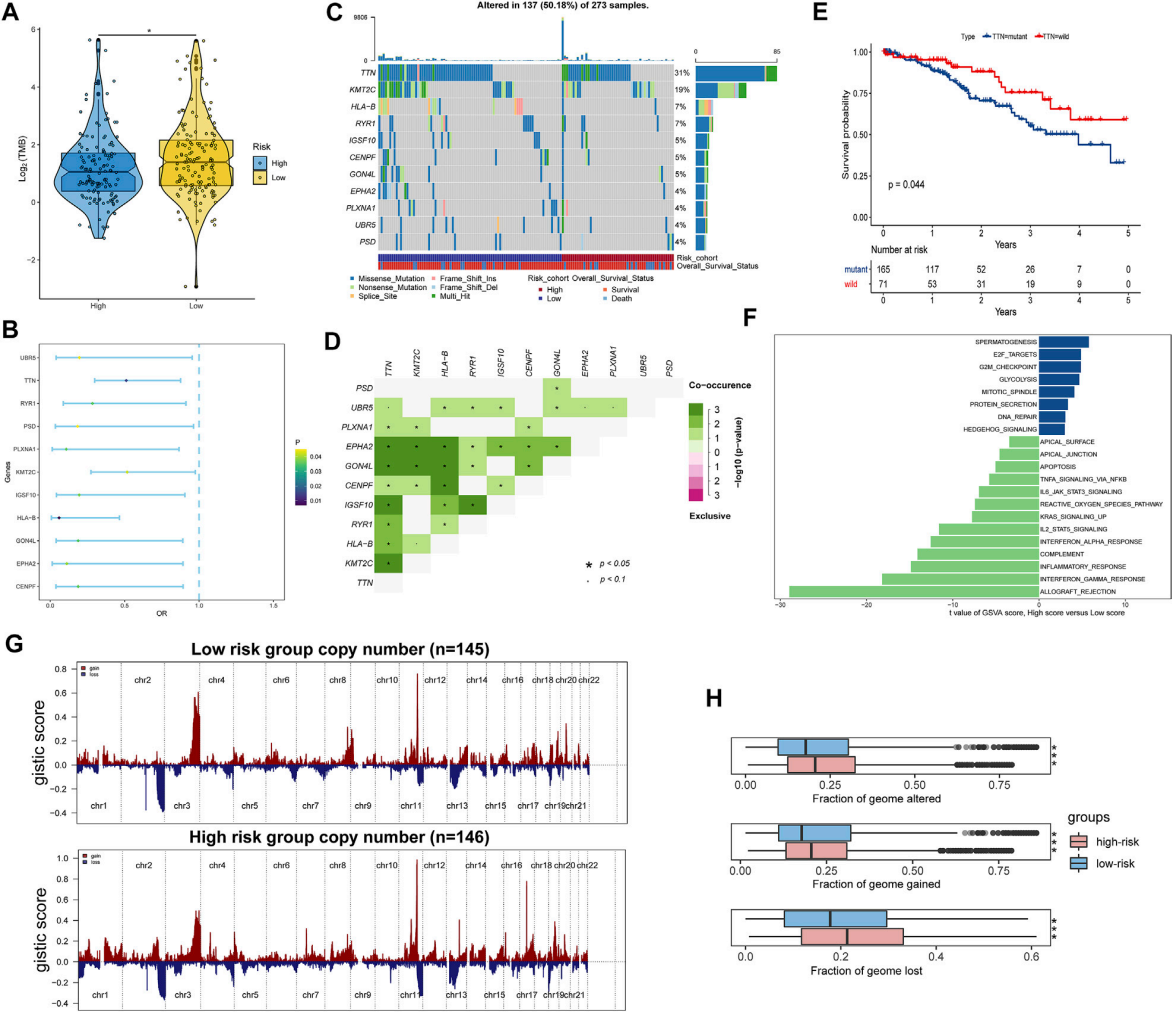
Identification differences of the genetic variation and pathway activation between high- and low-risk groups. **(A)** Tumor mutation burdens were compared among distinct risk groups. **(B)** Forest plot of genes with differences in mutation frequencies between the low- and high-risk groups. **(C)** Waterfall plot of the 11 mutant genes with significant frequency differences between low- and high-risk groups. **(D)** Interaction of differentially mutated genes. **(E)** Kaplan–Meier curve showing that patients with mutant *TTN* have a better OS than those with wild type. **(F)** Differential analysis of GSVA scores among distinct risk groups. **(G)** Copy number alteration gains (red) and losses (blue) between the low- and high-risk groups. **(H)** Differential analysis of altered, lost, and gained genome fractions (%) between the low-risk and high-risk groups. *p < 0.05; **p < 0.01; ***p < 0.001; ns, not significant.

Tumor mutation burden (TMB) is largely attributed to genomic instability and can indirectly reflect the ability and degree of tumor production of neoantigens and predict the immunotherapy efficacy of various tumors. We found that TMB was significantly higher in the low-risk score group than in the high-risk score group (Figure 7A). We further investigated the somatic mutations across CC patients. Logistic regression analysis showed that 11 genes mutation frequencies were significantly different between high- and low-risk score groups, including *CENPF*, *EPHA2*, *GON4L*, *HLA-B*, *IGSF10*, *KMT2C*, *PLXNA1*, *PSD*, *RYR1*, *TTN*, and *UBR5* (Figure 7B). The mutation frequencies of these genes are shown in Figure 7C, and there were significant co-occurrences among mutations of these genes (Figure 7D). We also found that patients with mutant *TTN* were significantly associated with better OS compared with wild-type patients (Figure 7E), suggesting that the *TTN* may be a potential immunotherapy target.

The GSVA also identified significant differences in biological functions between the high- and low-risk groups (Figure 7F, Supplementary Table S9). Consistent with the GSEA results, the direct comparison revealed that E2F targets, G2M checkpoint, glycolysis, and DNA repair pathways were significantly enriched in the high-risk group. Comparatively, apoptosis, KARS signaling up, and inflammatory response pathways were significantly enriched in the low-risk group. Subsequently, copy number variation analysis showed different patterns of chromosomal alteration between the high- and low-risk groups (Figure 7G). A larger proportion of genomic loss and gain were detected in the high-risk group (Figure 7H). Our analysis indicated that activation of tumor-related pathways, production of neoantigens, and amplification and deletion of certain tumor suppressor genes might cause differences in survival between high- and low-risk score groups.

### Identification of Potential Agents and Prediction of Immunotherapeutic Effect

**FIGURE 8:**
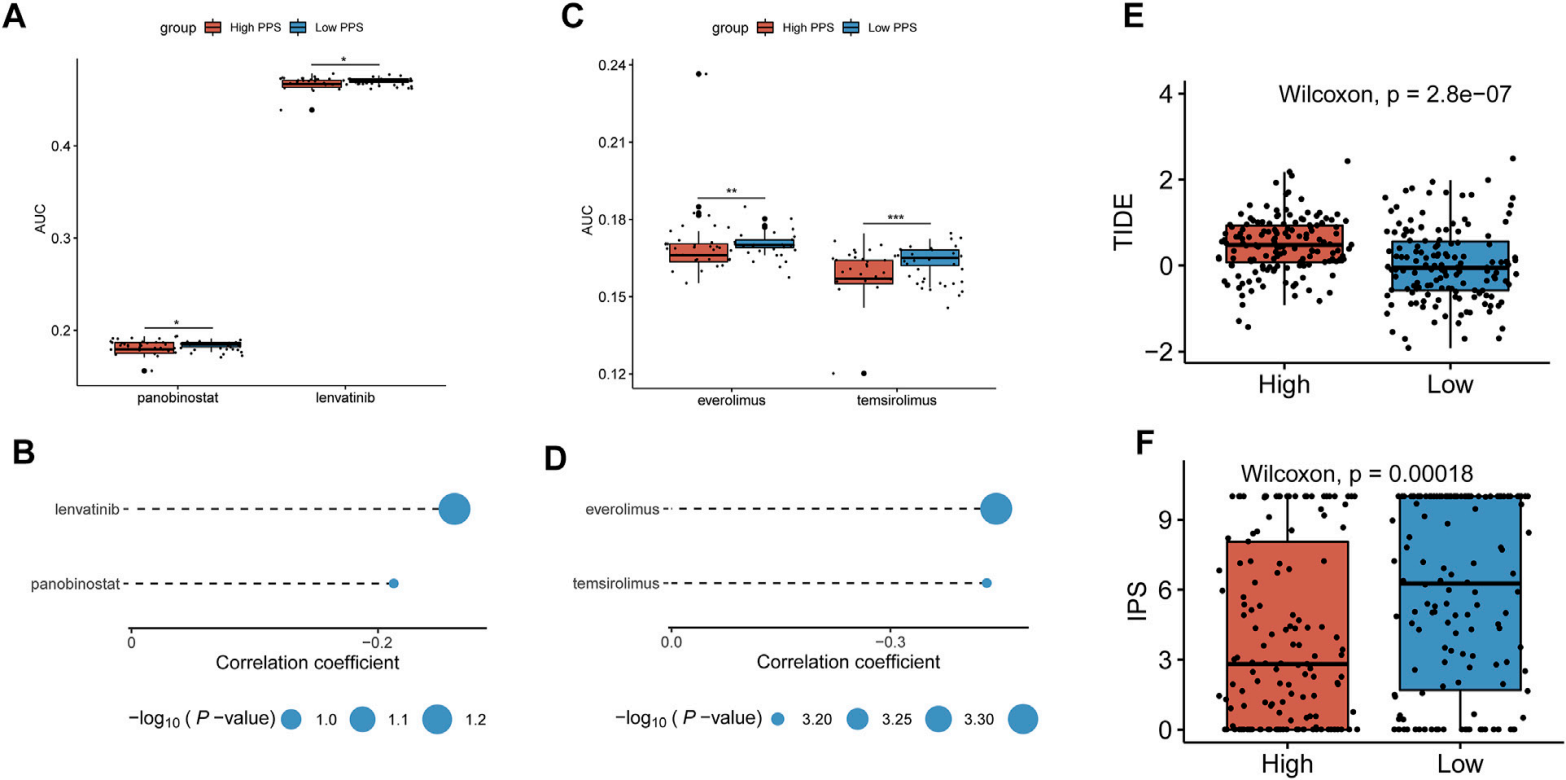
Identification of potential agents and prediction of immunotherapeutic effect. **(A,B)** Differential drug response analysis of the selected agents for CC patients between the higher and lower risk score groups based on the CTRP dataset and Spearman’s correlation analysis of CTRP-derived agents and risk score. **(C,D)** Differential drug response analysis of the selected agents for CC patients between the higher and lower risk score groups based on the PRISM dataset and Spearman’s correlation analysis of PRISM-derived agents and risk score. **(E,F)** The TIDE score and IPS were compared between the high- and low-risk groups. *p < 0.05; **p < 0.01; ***p < 0.001; ns, not significant.

Based on the CTRP and PRISM-derived drug response datasets, we used two approaches to identify potential agents for CC patients. First, we performed a differential drug response analysis between high-risk (upper decile) and low-risk (lower decile) groups to identify drugs with significantly different AUC values (log_2_FC > 0.01, *p* < 0.05). Next, the Spearman correlation between the risk score and the AUC value was conducted to screen out agents with a significantly negative correlation coefficient (r < −0.20 for CTRP and r < −0.40 for PRISM, *p* < 0.05). Finally, we determined two CTRP-derived compounds (panobinostat, lenvatinib) (Figures 8A,B) and two PRISM-derived compounds (everolimus, temsirolimus) (Figures 8C,D) as the potential agents for CC patients with high-risk scores. Moreover, we also calculated the TIDE score and IPS based on the TCGA gene expression profile to determine the immunotherapeutic response in CC patients. We found that patients in the low-risk group had lower TIDE scores and higher IPS (Figures 8E,F), suggesting that patients in the low-risk group were more likely to respond to immunotherapy than those in the high-risk group.

## Discussion

In this study, the ESTIMATE algorithm was performed to calculate the immune score and the stromal score to estimate the TME infiltration pattern of each CC patient in the TCGA cohort. Because the OS of patients in the high-immune group is better than that of patients in the low-immune group, the TME and DNA methylation-related genes were identified by the integrative analysis of DEGs and DMGs between the low- and high-immune score groups. Based on multiple LASSO Cox regression analysis, we constructed a nine-gene TME and DNA methylation-related prognostic signature to predict prognosis for stratified CC patients and performed external validation for its performance. Then, the signature was combined with the FIGO stage to generate a composite prognostic nomogram that reliably demonstrated the accurate prognosis prediction for patients with CC. Furthermore, we identified the tumor immune signature, function enrichment, genetic variants, and pathway activation associated with the prognostic signature. Finally, we predicted patients’ immunotherapy responses by the TIDE score and IPS and provided four potential agents for patients with high-risk scores.

The fundamental role of TME is the dynamic interaction of immune and stromal cells with malignant cells and can influence tumor growth, metastasis, and patient prognosis (Hanahan and Coussens, 2012). Many epigenetic studies have shown that DNA methylation plays a key role in promoting cellular responses to stimuli and regulating immune cell differentiation (Sørensen et al., 2010; Smith and Meissner, 2013). Thus, it is generally accepted that DNA methylation has a very complex regulatory role on the TME, especially during the development of immune and stromal cells. For example, one study has found that different methylation patterns exist in myeloid and lymphoid lineages in cancer tissues. During the differentiation and activation of macrophages, the global methylation level increased, while it decreased in both T and B lymphocytes (Schuyler et al., 2016). Most importantly, DNA methylation can influence not only the expression levels of genes important for immune cell development but also the tumor immune response in the TME. One study suggested that Th1/Th2 differentiation may be mediated by methylation and demethylation of the FN-γ in naive CD4^+^ T lymphocytes (Janson et al., 2008). Another report revealed that hypermethylation of genes (*LAX1*, *SIT1*, and *UBASH3A*) leads to enhanced anti-tumor T-cell responses in breast cancer (Dedeurwaerder et al., 2011). Moreover, a previous study showed that in non-small-cell lung cancer, demethylation of the *FOXP3* gene promoter could reduce the activity of DNMTs in Tregs CD4^+^ lymphocytes and downregulate immune responses in the TME (Ke et al., 2016).

In the present study, we have observed that CC patients with low immunity levels have worse survival than those with high immunity levels, which may be due to a decrease in the immune infiltration levels caused by hypermethylation in the promoter region of immune-related genes affecting gene expression levels. To predict CC patient survival, we constructed a nine-gene TME and DNA methylation-related prognostic signature. In the training and validation datasets, the risk score of the signature was an independent prognosis factor and had a good predictive effect. Among the nine genes included in the signature, their coded proteins correlate with the immune system, such as *CCR7*, which coded protein belonging to the CCR7 chemokine axis. The axis is involved in the trafficking of effector cells for many immune responses and controls the migration and metastasis of tumor cells to the lymphatic system (Salem et al., 2021). CD6 is one kind of type I transmembrane glycoprotein on the lymphocyte surface and is involved in the development and differentiation of lymphocytes (Santos et al., 2016). As a B cell transcriptional coactivator, POU2AF1 regulates the expression of B cell maturation factor TNFRSF17 and stimulates the growth of myeloma cells (Zhao et al., 2008). DNASE1L3 is a kind of deoxyribonuclease and is involved in neutrophil activation and acute inflammatory responses (Jiménez-Alcázar et al., 2017). IL12RB2 is the interleukin-12 receptor. A study found that *IL12RB2* knockout (KO) mice develop autoimmunity, lymphoid proliferation, and B-cell tumors and suggested IL12RB2 functions physiologically in inhibiting aberrant B-cell activation (Airoldi et al., 2005). Moreover, we established a composite nomogram based on the FIGO stage and the signature to guide the prognosis prediction of CC patients more effectively. The composite nomogram demonstrated higher accuracy of prognosis and greater net benefits than the FIGO stage and the signature.

Furthermore, our study results showed that the stromal and immune scores were negatively correlated with the risk score, and patients in the high-risk group had lower immune scores and were more likely to be immunosuppressed. More seriously, patients in the high-risk group had a lower immune activity, including lower immune cell infiltration such as T cell CD4^+^, T cell CD8^+^, and downregulation of HLA family genes and immune checkpoints expression such as *HLA-A*, *HLA-B*, *PD1*, and *CTLA4*, which contributed to immunosuppression and tumor immune escape. We further analyzed GSEA pathway enrichment in high- and low-risk groups and found that proliferation-specific pathways were significantly enriched in the high-risk group, such as the Myc targets v1 and E2F targets pathway, while apoptosis, KRAS signaling up, and inflammatory response pathway were significantly enriched in the low-risk group.

Compared to other malignancies, immunotherapy plays an even more important role in cervical cancer. For example, in precancerous abnormalities and early tumors of cervical cancer, restoring the immune response to cancer cells and strengthening immune system function to HPV may stop further progression (Lee et al., 2016). TMB measures the number of nonsynonymous mutations of cancers, and more mutations could generate more neo-antigens, thereby activating the patient’s immune system and benefiting cancer immunotherapy (Jardim et al., 2021). Therefore, many studies have suggested that TMB could be a good predictive biomarker of immunotherapy response (Chalmers et al., 2017; Büttner et al., 2019). We found that patients in the low-risk group had higher TMB than those in the high-risk group. Taken together, the results of TMB, IPS, and TIDE scores suggested that patients with lower risk scores may benefit more from immunotherapy. In addition, somatic mutations analysis revealed that the mutation frequency of 11 genes was significantly different between the high- and low-risk groups. There were co-mutations in these genes, suggesting that they may synergistically affect the regulation of TME. Interestingly, patients with mutant *TTN* had better OS than those with the wild type and *TTN* may be a potential immunotherapy target. We also determined that the genetic variants were significantly different between the high- and low-risk groups. The high-risk group had a significantly higher fraction of genome altered than the low-risk group, indicating that patients with high-risk scores had more unstable genomes, and some tumor-promoting pathways were activated, leading to poor prognosis.

Immunotherapy has a demonstrable synergistic activity to alter or enhance the immune system when combined with radiotherapy, chemoradiotherapy, and targeted drugs (Dyer et al., 2021). To identify drugs that synergize with immunotherapy for high-risk patients and facilitate personalized treatment decisions, we identified four potential agents for high-risk CC patients by interaction analysis between the risk signature and drug responses. Among the four candidate agents, lenvatinib is a multikinase inhibitor of receptor tyrosine kinases. Panobinostat is a nonselective HDAC inhibitor. Both everolimus and temsirolimus are inhibitors of mTOR kinase, which is part of the signaling pathway associated with cell growth and proliferation. Many studies have found that the destruction of mTOR leads to the suppression of the cell cycle and angiogenesis, thereby inhibiting the development of cervical cancer. These studies also validated the reliability of our results (Bossler et al., 2019; Sun et al., 2020; Yang et al., 2020).

Our study has its limitations. First, although our signature is beneficial in evaluating prognosis and conducting therapies for CC patients, it does not yield a satisfactory result in the validation set as their patients are in the early stage of CC. It should be prospectively validated in other datasets. Second, because there are no expression data for CC patients receiving immunotherapy, we only used bioinformatics analysis to predict the effect of immunotherapy in CC patients in the TCGA dataset, and there is no actual immunotherapy benefit of immunotherapy for patients with different risk scores. Third, drug clinical trials and experimental exploration are needed to validate our drug prediction results. In summary, our study highlights the value of the TME and DNA methylation-related signature in predicting prognosis and immune response.

## Data Availability

The datasets presented in this study can be found in online repositories. The names of the repository/repositories and accession number(s) can be found in the article/Supplementary Material.

## References

[B1] Airoldi I. Di Carlo E. Cocco C. Sorrentino C. Fais F. Cilli M. (2005). Lack of Il12rb2 Signaling Predisposes to Spontaneous Autoimmunity and Malignancy. Blood 106 (12), 3846–3853. 10.1182/blood-2005-05-2034 16081683

[B2] Ali M. A. Matboli M. Tarek M. Reda M. Kamal K. M. Nouh M. (2017). Epigenetic Regulation of Immune Checkpoints: Another Target for Cancer Immunotherapy? Immunotherapy 9 (1), 99–108. 10.2217/imt-2016-0111 28000527

[B3] Anghel C. V. Quon G. Haider S. Nguyen F. Deshwar A. G. Morris Q. D. (2015). ISOpureR: an R Implementation of a Computational Purification Algorithm of Mixed Tumour Profiles. BMC Bioinformatics 16, 156. 10.1186/s12859-015-0597-x 25972088 PMC4429941

[B4] Becht E. Giraldo N. A. Lacroix L. Buttard B. Elarouci N. Petitprez F. (2016). Estimating the Population Abundance of Tissue-Infiltrating Immune and Stromal Cell Populations Using Gene Expression. Genome Biol. 17 (1), 218. 10.1186/s13059-016-1070-5 27765066 PMC5073889

[B5] Binnewies M. Roberts E. W. Kersten K. Chan V. Fearon D. F. Merad M. (2018). Understanding the Tumor Immune Microenvironment (TIME) for Effective Therapy. Nat. Med. 24 (5), 541–550. 10.1038/s41591-018-0014-x 29686425 PMC5998822

[B6] Bird A. (2007). Perceptions of Epigenetics. Nature 447 (7143), 396–398. 10.1038/nature05913 17522671

[B7] Bossler F. Hoppe-Seyler K. Hoppe-Seyler F. (2019). PI3K/AKT/mTOR Signaling Regulates the Virus/Host Cell Crosstalk in HPV-Positive Cervical Cancer Cells. Int. J. Mol. Sci. 20 (9), 2188. 10.3390/ijms20092188 PMC6539191 31058807

[B8] Büttner R. Longshore J. W. López-Ríos F. Merkelbach-Bruse S. Normanno N. Rouleau E. (2019). Implementing TMB Measurement in Clinical Practice: Considerations on Assay Requirements. ESMO Open 4 (1), e000442. 10.1136/esmoopen-2018-000442 30792906 PMC6350758

[B9] Chalmers Z. R. Connelly C. F. Fabrizio D. Gay L. Ali S. M. Ennis R. (2017). Analysis of 100,000 Human Cancer Genomes Reveals the Landscape of Tumor Mutational burden. Genome Med. 9 (1), 34. 10.1186/s13073-017-0424-2 28420421 PMC5395719

[B10] Charoentong P. Finotello F. Angelova M. Mayer C. Efremova M. Rieder D. (2017). Pan-cancer Immunogenomic Analyses Reveal Genotype-Immunophenotype Relationships and Predictors of Response to Checkpoint Blockade. Cel Rep. 18 (1), 248–262. 10.1016/j.celrep.2016.12.019 28052254

[B11] Cohen P. A. Jhingran A. Oaknin A. Denny L. (2019). Cervical Cancer. Lancet 393 (10167), 169–182. 10.1016/S0140-6736(18)32470-X 30638582

[B12] De Simone M. Arrigoni A. Rossetti G. Gruarin P. Ranzani V. Politano C. (2016). Transcriptional Landscape of Human Tissue Lymphocytes Unveils Uniqueness of Tumor-Infiltrating T Regulatory Cells. Immunity 45 (5), 1135–1147. 10.1016/j.immuni.2016.10.021 27851914 PMC5119953

[B13] Dedeurwaerder S. Desmedt C. Calonne E. Singhal S. K. Haibe‐Kains B. Defrance M. (2011). DNA Methylation Profiling Reveals a Predominant Immune Component in Breast Cancers. EMBO Mol. Med. 3 (12), 726–741. 10.1002/emmm.201100801 21910250 PMC3377115

[B14] Dyer B. A. Feng C. H. Eskander R. Sharabi A. B. Mell L. K. McHale M. (2021). Current Status of Clinical Trials for Cervical and Uterine Cancer Using Immunotherapy Combined with Radiation. Int. J. Radiat. Oncol. Biol. Phys. 109 (2), 396–412. 10.1016/j.ijrobp.2020.09.016 32942005

[B15] Easwaran H. Tsai H.-C. Baylin S. B. (2014). Cancer Epigenetics: Tumor Heterogeneity, Plasticity of Stem-like States, and Drug Resistance. Mol. Cel 54 (5), 716–727. 10.1016/j.molcel.2014.05.015 PMC4103691 24905005

[B16] Fu J. Li K. Zhang W. Wan C. Zhang J. Jiang P. (2020). Large-scale Public Data Reuse to Model Immunotherapy Response and Resistance. Genome Med. 12 (1), 21. 10.1186/s13073-020-0721-z 32102694 PMC7045518

[B17] Ghandi M. Huang F. W. Jané-Valbuena J. Kryukov G. V. Lo C. C. McDonald E. R. (2019). Next-generation Characterization of the Cancer Cell Line Encyclopedia. Nature 569 (7757), 503–508. 10.1038/s41586-019-1186-3 31068700 PMC6697103

[B18] Hanahan D. Coussens L. M. (2012). Accessories to the Crime: Functions of Cells Recruited to the Tumor Microenvironment. Cancer Cell 21 (3), 309–322. 10.1016/j.ccr.2012.02.022 22439926

[B19] Herrero R. González P. Markowitz L. E. (2015). Present Status of Human Papillomavirus Vaccine Development and Implementation. Lancet Oncol. 16 (5), e206–e216. 10.1016/S1470-2045(14)70481-4 25943065

[B20] Janson P. C. J. Marits P. Thörn M. Ohlsson R. Winqvist O. (2008). CpG Methylation of the IFNG Gene as a Mechanism to Induce Immunosupression in Tumor-Infiltrating Lymphocytes. J. Immunol. 181 (4), 2878–2886. 10.4049/jimmunol.181.4.2878 18684979

[B21] Jardim D. L. Goodman A. de Melo Gagliato D. Kurzrock R. (2021). The Challenges of Tumor Mutational Burden as an Immunotherapy Biomarker. Cancer Cell 39 (2), 154–173. 10.1016/j.ccell.2020.10.001 33125859 PMC7878292

[B22] Jiao Y. Widschwendter M. Teschendorff A. E. (2014). A Systems-Level Integrative Framework for Genome-wide DNA Methylation and Gene Expression Data Identifies Differential Gene Expression Modules under Epigenetic Control. Bioinformatics 30 (16), 2360–2366. 10.1093/bioinformatics/btu316 24794928

[B23] Jiménez-Alcázar M. Rangaswamy C. Panda R. Bitterling J. Simsek Y. J. Long A. T. (2017). Host DNases Prevent Vascular Occlusion by Neutrophil Extracellular Traps. Science 358 (6367), 1202–1206. 10.1126/science.aam8897 29191910

[B24] Johnston R. J. Su L. J. Pinckney J. Critton D. Boyer E. Krishnakumar A. (2019). VISTA Is an Acidic pH-Selective Ligand for PSGL-1. Nature 574 (7779), 565–570. 10.1038/s41586-019-1674-5 31645726

[B25] Ke X. Zhang S. Xu J. Liu G. Zhang L. Xie E. (2016). Non-small-cell Lung Cancer-Induced Immunosuppression by Increased Human Regulatory T Cells via Foxp3 Promoter Demethylation. Cancer Immunol. Immunother. 65 (5), 587–599. 10.1007/s00262-016-1825-6 27000869 PMC11028464

[B26] Langfelder P. Horvath S. (2008). WGCNA: an R Package for Weighted Correlation Network Analysis. BMC Bioinformatics 9, 559. 10.1186/1471-2105-9-559 19114008 PMC2631488

[B27] Lee S.-J. Yang A. Wu T.-C. Hung C.-F. (2016). Immunotherapy for Human Papillomavirus-Associated Disease and Cervical Cancer: Review of Clinical and Translational Research. J. Gynecol. Oncol. 27 (5), e51. 10.3802/jgo.2016.27.e51 27329199 PMC4944018

[B28] Li B. Severson E. Pignon J.-C. Zhao H. Li T. Novak J. (2016). Comprehensive Analyses of Tumor Immunity: Implications for Cancer Immunotherapy. Genome Biol. 17 (1), 174. 10.1186/s13059-016-1028-7 27549193 PMC4993001

[B29] Lin K. Roosinovich E. Ma B. Hung C.-F. Wu T.-C. (2010). Therapeutic HPV DNA Vaccines. Immunol. Res. 47 (1-3), 86–112. 10.1007/s12026-009-8141-6 20066511 PMC2891127

[B30] Mayakonda A. Lin D.-C. Assenov Y. Plass C. Koeffler H. P. (2018). Maftools: Efficient and Comprehensive Analysis of Somatic Variants in Cancer. Genome Res. 28 (11), 1747–1756. 10.1101/gr.239244.118 30341162 PMC6211645

[B31] Newman A. M. Liu C. L. Green M. R. Gentles A. J. Feng W. Xu Y. (2015). Robust Enumeration of Cell Subsets from Tissue Expression Profiles. Nat. Methods 12 (5), 453–457. 10.1038/nmeth.3337 25822800 PMC4739640

[B32] Ogilvie G. S. van Niekerk D. Krajden M. Smith L. W. Cook D. Gondara L. (2018). Effect of Screening with Primary Cervical HPV Testing vs Cytology Testing on High-Grade Cervical Intraepithelial Neoplasia at 48 Months. JAMA 320 (1), 43–52. 10.1001/jama.2018.7464 29971397 PMC6583046

[B33] Piao Y. Piao M. Park K. Ryu K. H. (2012). An Ensemble Correlation-Based Gene Selection Algorithm for Cancer Classification with Gene Expression Data. Bioinformatics 28 (24), 3306–3315. 10.1093/bioinformatics/bts602 23060613

[B34] Ritchie M. E. Phipson B. Wu D. Hu Y. Law C. W. Shi W. (2015). Limma powers Differential Expression Analyses for RNA-Sequencing and Microarray Studies. Nucleic Acids Res. 43 (7), e47. 10.1093/nar/gkv007 25605792 PMC4402510

[B35] Salem A. Alotaibi M. Mroueh R. Basheer H. A. Afarinkia K. (2021). CCR7 as a Therapeutic Target in Cancer. Biochim. Biophys. Acta Rev. Cancer 1875 (1), 188499. 10.1016/j.bbcan.2020.188499 33385485

[B36] Santos R. F. Oliveira L. M. Carmo A. (2016). Tuning T Cell Activation: The Function of CD6 at the Immunological Synapse and in T Cell Responses. Curr. Drug Targets 17 (6), 630–639. 10.2174/1389450116666150531152439 26028048

[B37] Schuyler R. P. Merkel A. Raineri E. Altucci L. Vellenga E. Martens J. H. A. (2016). Distinct Trends of DNA Methylation Patterning in the Innate and Adaptive Immune Systems. Cel Rep. 17 (8), 2101–2111. 10.1016/j.celrep.2016.10.054 PMC5889099 27851971

[B38] Sharma S. Kelly T. K. Jones P. A. (2010). Epigenetics in Cancer. Carcinogenesis 31 (1), 27–36. 10.1093/carcin/bgp220 19752007 PMC2802667

[B39] Small W. Bacon M. A. Bajaj A. Chuang L. T. Fisher B. J. Harkenrider M. M. (2017). Cervical Cancer: A Global Health Crisis. Cancer 123 (13), 2404–2412. 10.1002/cncr.30667 28464289

[B40] Smith Z. D. Meissner A. (2013). DNA Methylation: Roles in Mammalian Development. Nat. Rev. Genet. 14 (3), 204–220. 10.1038/nrg3354 23400093

[B41] Sørensen A. L. Timoskainen S. West F. D. Vekterud K. Boquest A. C. Ährlund-Richter L. (2010). Lineage-specific Promoter DNA Methylation Patterns Segregate Adult Progenitor Cell Types. Stem Cell Develop. 19 (8), 1257–1266. 10.1089/scd.2009.0309 19886822

[B42] Subramanian A. Tamayo P. Mootha V. K. Mukherjee S. Ebert B. L. Gillette M. A. (2005). Gene Set Enrichment Analysis: a Knowledge-Based Approach for Interpreting Genome-wide Expression Profiles. Proc. Natl. Acad. Sci. U.S.A. 102 (43), 15545–15550. 10.1073/pnas.0506580102 16199517 PMC1239896

[B43] Sun X. Shu Y. Xu M. Jiang J. Wang L. Wang J. (2020). ANXA6 Suppresses the Tumorigenesis of Cervical Cancer through Autophagy Induction. Clin. Transl. Med. 10 (6), e208. 10.1002/ctm2.208 33135350 PMC7571625

[B44] Wakeham K. Kavanagh K. (2014). The burden of HPV-Associated Anogenital Cancers. Curr. Oncol. Rep. 16 (9), 402. 10.1007/s11912-014-0402-4 25118645

[B45] Wendel Naumann R. Leath C. A. (2020). Advances in Immunotherapy for Cervical Cancer. Curr. Opin. Oncol. 32 (5), 481–487. 10.1097/CCO.0000000000000663 32740092 PMC7748319

[B46] Wright J. D. Matsuo K. Huang Y. Tergas A. I. Hou J. Y. Khoury-Collado F. (2019). Prognostic Performance of the 2018 International Federation of Gynecology and Obstetrics Cervical Cancer Staging Guidelines. Obstet. Gynecol. 134 (1), 49–57. 10.1097/AOG.0000000000003311 31188324 PMC7641496

[B47] Yang Y. Wang Q. Song D. Zen R. Zhang L. Wang Y. (2020). Lysosomal Dysfunction and Autophagy Blockade Contribute to Autophagy-Related Cancer Suppressing Peptide-Induced Cytotoxic Death of Cervical Cancer Cells through the AMPK/mTOR Pathway. J. Exp. Clin. Cancer Res. 39 (1), 197. 10.1186/s13046-020-01701-z 32962728 PMC7510096

[B48] Yang C. Huang X. Li Y. Chen J. Lv Y. Dai S. (2021). Prognosis and Personalized Treatment Prediction in TP53-Mutant Hepatocellular Carcinoma: an In Silico Strategy towards Precision Oncology. Brief Bioinform. 22 (3), bbaa164. 10.1093/bib/bbaa164 32789496

[B49] Yoshihara K. Shahmoradgoli M. Martínez E. Vegesna R. Kim H. Torres-Garcia W. (2013). Inferring Tumour Purity and Stromal and Immune Cell Admixture from Expression Data. Nat. Commun. 4, 2612. 10.1038/ncomms3612 24113773 PMC3826632

[B50] Zhao C. Inoue J. Imoto I. Otsuki T. Iida S. Ueda R. (2008). POU2AF1, an Amplification Target at 11q23, Promotes Growth of Multiple Myeloma Cells by Directly Regulating Expression of a B-Cell Maturation Factor, TNFRSF17. Oncogene 27 (1), 63–75. 10.1038/sj.onc.1210637 17621271

