# Supplementary material for: Identification of Tumor Microenvironment and DNA Methylation-Related Prognostic Signature for Predicting Clinical Outcomes and Therapeutic Responses in Cervical Cancer
Source: Front Mol Biosci. 2022 Apr 19;9:872932. doi: 10.3389/fmolb.2022.872932 (PMC9061945; doi:10.3389/fmolb.2022.872932)
Supplement: Supplementary file 1 [file DataSheet1.docx]

**
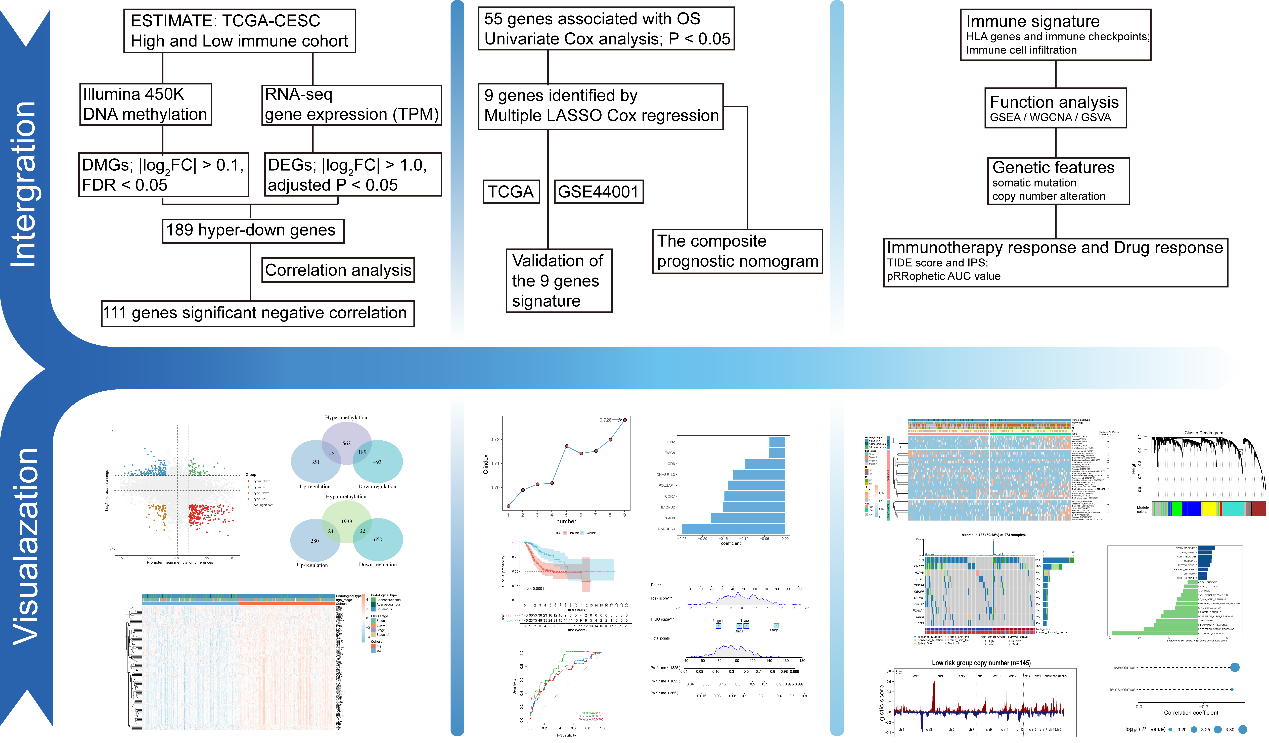
**

**Supplementary Figure S1 |** The detailed workflow of the study.

**
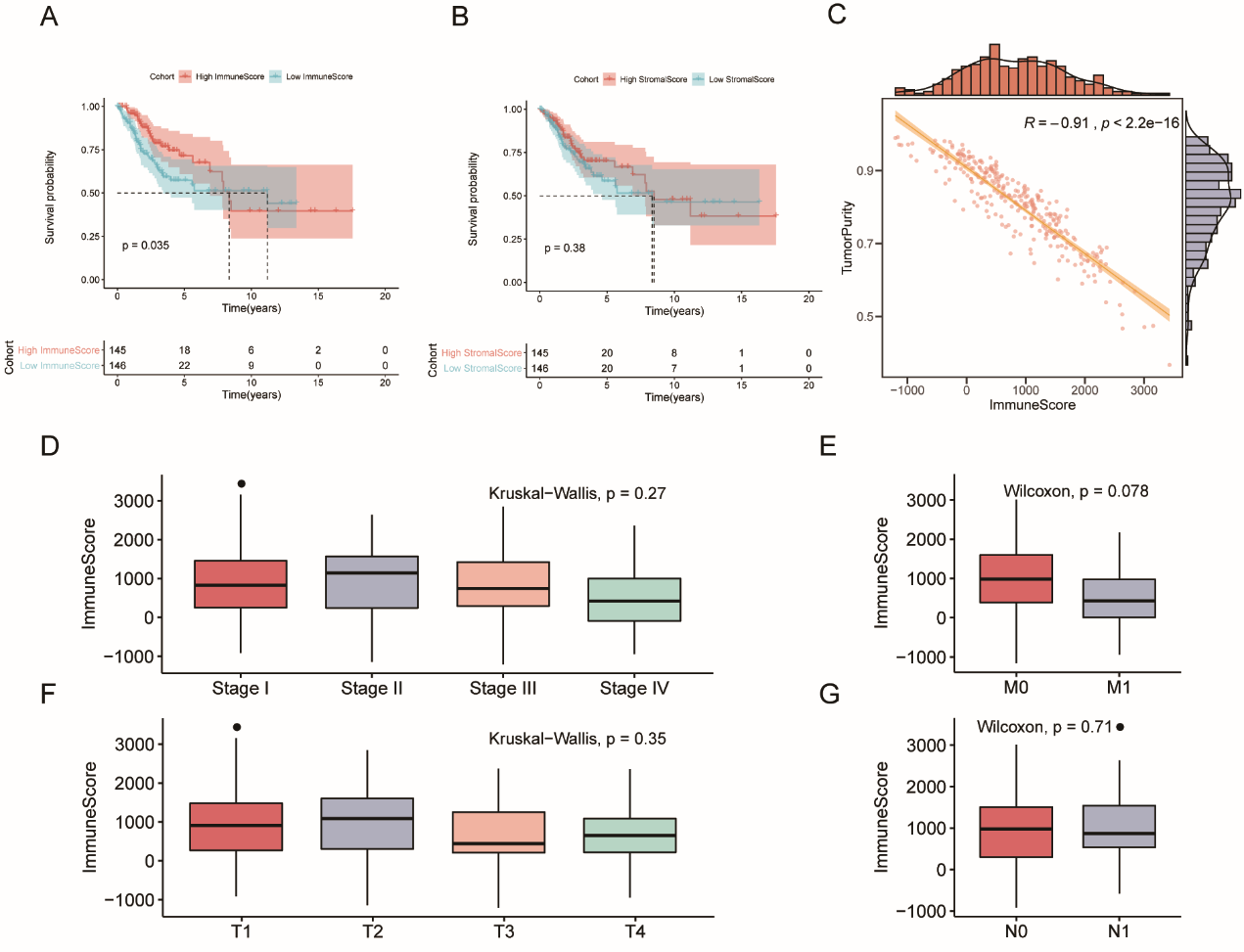
**

**Supplemental Figure** **S2 |** Correlation between immune score and clinical information. (**A**) Kaplan-Meier curve shows that OS was significantly different between the high-immune and low-immune groups. (**B**) Kaplan-Meier curve shows that there was no significant difference in OS between the high- and low-stromal groups. (**C**) Correlation analysis of the immune score and tumor purity of CC patients. (**D–G**) Difference analysis of the distribution of immune score in different FIGO stage and TNM stage.

**
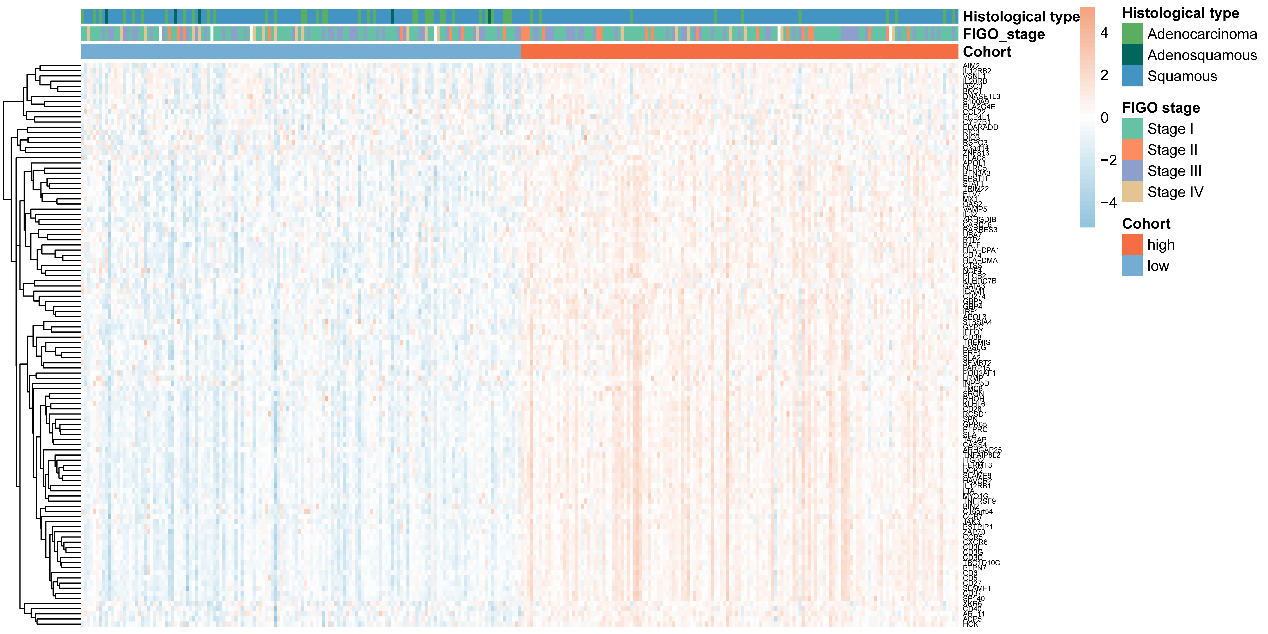
**

**Supplementary Figure S3 |** Heat map of genes which expression levels are significantly negatively correlated with DNA promoter mean methylation.

**
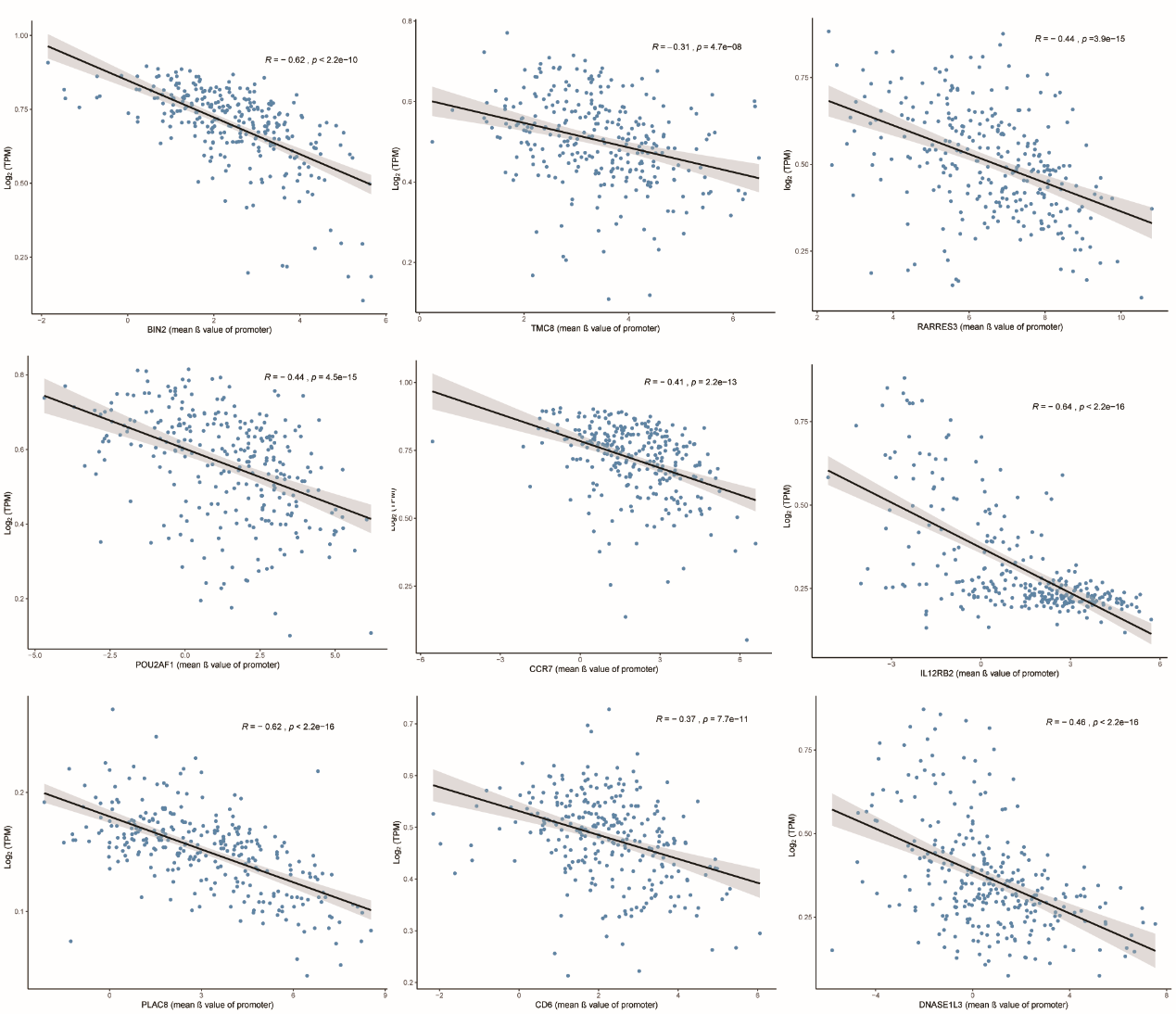
**

**Supplementary Figure S4 |** The correlation between prognosis-associated genes expression and DNA promoter mean methylation level in TCGA cohort.

**
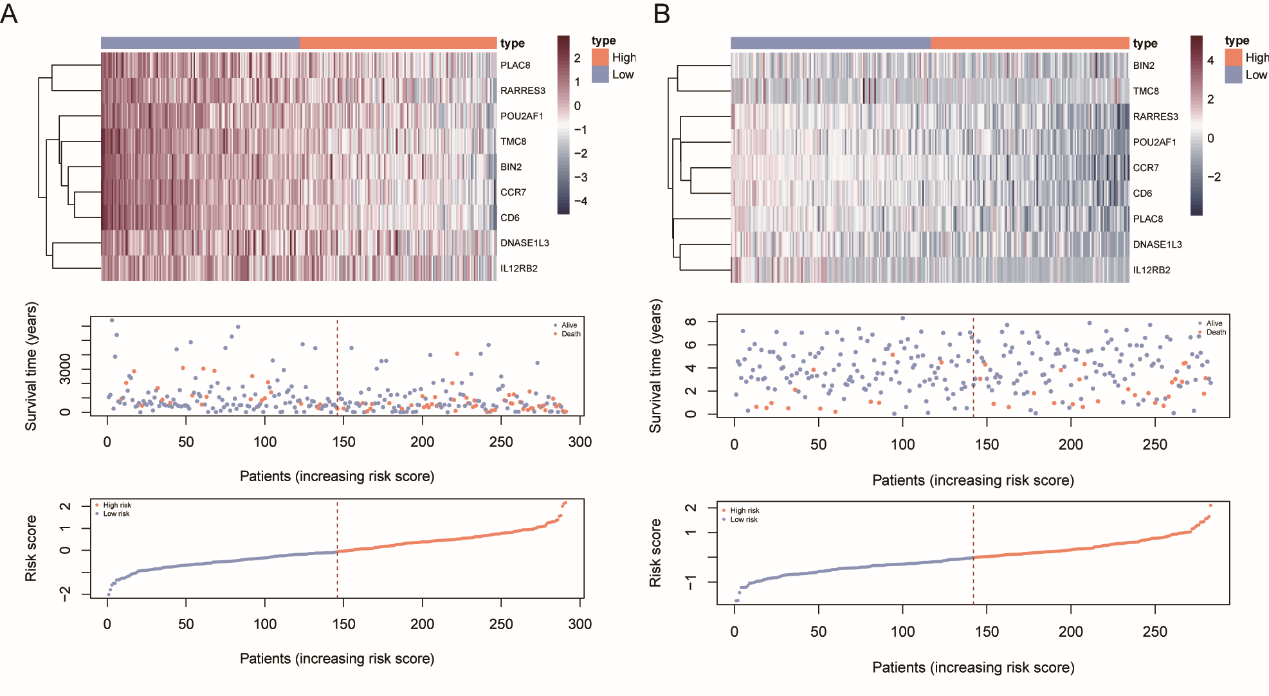
****Supplementary Figure S5 |** Heatmaps of the signature consisting of 9 prognosis-associated genes and the risk score curve. TCGA (**A**) and GSE44001 (**B**) cohorts.

**
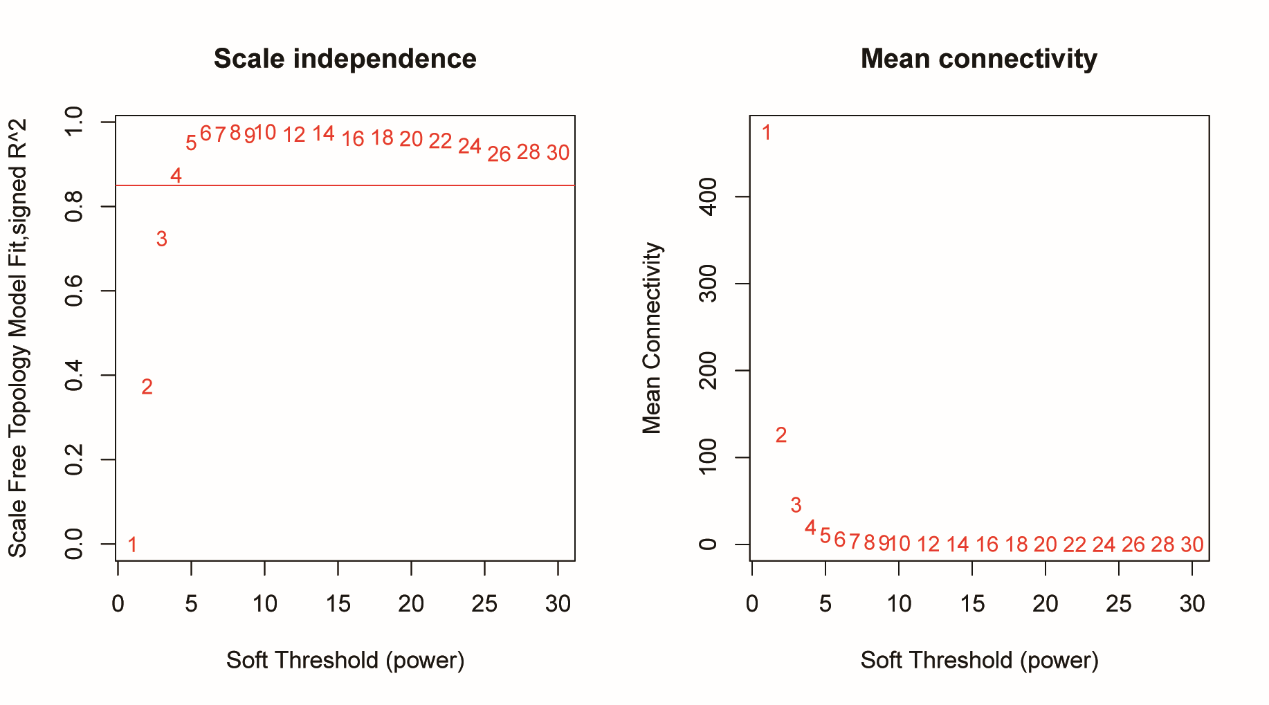
****Supplementary Figure S6 |** Identification of the soft threshold according to the standard of the scale-free network. The red line represents the threshold line of 0.85.
